# The effects of vitamin and mineral supplementation on women with gestational diabetes mellitus

**DOI:** 10.1186/s12902-021-00712-x

**Published:** 2021-05-24

**Authors:** Dandan Li, Zixin Cai, Zhenhong Pan, Yan Yang, Jingjing Zhang

**Affiliations:** National Clinical Research Center for Metabolic Diseases, Metabolic Syndrome Research Center, Key Laboratory of Diabetes Immunology (Central South University), Ministry of Education, and Department of Metabolism and Endocrinology, The Second Xiangya Hospital of Central South University, Changsha 410011, Hunan China

**Keywords:** Gestational diabetes mellitus, Vitamins, Minerals, Glycemic control, Inflammation, Oxidative stress

## Abstract

**Background:**

The effects of vitamin and mineral supplementation on women with gestational diabetes mellitus (GDM) have not been well established. We conduct a meta-analysis to evaluate the effects of vitamin and mineral supplementation on glycemic control, inflammation and oxidative stress for women with GDM.

**Methods:**

A systematic search of randomized controlled trials (RCTs) was conducted from PubMed, Embase, Web of Science and Cochrane Library up to July, 2020. Various results were pooled by using Review manager 5.3 and Stata 12.0. Mean difference (MD) with 95% confidence interval (CI) was estimated. Heterogeneity between studies was assessed by I-squared (*I*^*2*^) tests.

**Results:**

Six hundred ninety-eight patients from 12 trials were included in our meta-analysis. Magnesium, zinc, selenium, calcium, vitamin D and E (alone or in combination) were found to significantly improve glycemic control in women with GDM compared to those receiving placebos: fasting plasma glucose (FPG) (MD = - 9.02; 95% CI: - 12.09, - 5.96; *P* < 0.00001), serum insulin (MD = - 4.33; 95% CI: - 5.35, - 3.32; *P* < 0.00001), homeostasis model assessment-insulin resistance (HOMA-IR) (MD = - 1.34; 95% CI: - 1.60, - 1.07; *P* < 0.00001), and homeostasis model of assessment for β cell function (HOMA-B) (MD = - 15.58; 95% CI: - 23.70, - 7.46; *P* = 0.0002). Vitamin and mineral supplementation was found to attenuated inflammation and oxidative stress through decreasing high-sensitivity C-reactive protein (hs-CRP) (MD = - 1.29; 95% CI: - 1.82, - 0.76; *P* < 0.00001), malondialdehyde (MDA) (MD = - 0.71; 95% CI: - 0.97, - 0.45; *P* < 0.00001), and increasing total antioxidant capacity (TAC) (MD = 45.55; 95% CI: 22.02, 69.08; *P* = 0.0001).

**Conclusions:**

This meta-analysis shows that vitamin and mineral supplementation significantly improved glycemic control, attenuated inflammation and oxidative stress in women with GDM.

**Supplementary Information:**

The online version contains supplementary material available at 10.1186/s12902-021-00712-x.

## Background

Gestational diabetes mellitus (GDM) is defined as impaired glucose tolerance with onset or first recognition during pregnancy [1]. Many risk factors, including obesity, gestational age and genetic background, contribute to the development of GDM [2]. The prevalence of GDM was reported approximately 15 to 20% worldwide [3]. Hyperglycemia during pregnancy is associated with adverse outcomes of both mother and offspring [4]. Women with a history of GDM are at increased risk of developing insulin resistance syndrome (IRS) and cardiovascular disease (CVD) later in life [4, 5].

Chronic low-grade inflammation is related to many known risk factors of GDM [6]. Increased degrees of inflammation during early pregnancy are associated with increased risk of GDM and the development of hyperglycemia [7]. In addition, the presence of oxidative stress has been reported in GDM [8–10], and the antioxidant status in women with GDM was down-regulated [11]. Oxidative stress plays important roles both in the pathogenesis and complications of GDM, supplementary therapy with antioxidants might help to reverse the oxidative status and improve the neonatal outcome [12, 13]. Zinc deficiency [14], hypomagnesemia [15] are common features in diabetes, and low circulating levels of vitamin D [16], vitamin E [11], and magnesium [17] have been found in women with GDM. Many reports showed that vitamin and mineral supplementation, such as vitamin D [18], vitmin E [13], magnesium [15, 19], and selenium [20] may regulate glucose metabolism and have beneficial roles in anti-inflammatory and anti-oxidative stress.

Nowadays, there is a growing interest to use vitamin and mineral supplementation during pregnancy, especially for women with GDM. Vitamin D is the most commonly used nutrient in the treatment of GDM, but the effect of vitamin D supplementation on glycemic control in GDM remains controversial. Some studies showed that vitamin D supplementation had beneficial effects on glycemia in women with GDM [18]. However, other studies showed that vitamin D supplementation had no significant effect on fasting plasma glucose (FPG) or fasting blood glucose (FBG) level in patients with GDM [21]. The role of selenium in glucose control in GDM was also inconsistent. A clinical trial showed that 200 μg selenium supplements for 6 weeks significantly reduced FPG and serum insulin levels in GDM patients [20]. However, another clinical trial showed that 100 μg selenium supplements for 12 weeks has no significant effect in regulating glucose homeostasis in women with GDM [22]. Studies on the effects of vitamin D and other nutritional supplementation on biomarkers of inflammation and oxidative stress in patients with GDM also have inconsistent results [18, 23, 24]. It was reported that vitamin D supplementation had no significant effect on hs-CRP, plasma TAC, and total GSH [18]. However, other studies showed that vitamin D or vitamin D-calcium co-supplementation had beneficial effects on biomarkers of inflammation and oxidative stress [23, 24]. Thus, this meta-analysis was conducted to evaluate the effects of vitamin and mineral supplementation, especially vitamin and mineral co-supplementation, on glycemic control, inflammation and oxidative stress in GDM patients.

## Methods

### Search strategy and study selection

This systematic literature search was done according to the Preferred Reporting Items for Systematic Reviews and Meta-Analyses (PRISMA) guidelines [25]. PubMed, Embase, Web of Science and Cochrane Library of RCTs were searched up to July, 2020. Search terms include Medical Subject Headings (MeSH) and keywords related to GDM, vitamins, minerals, micronutrients, glycemic control, inflammation and oxidative stress. The details of the search strategy are reported in the supplementary information. The search was limited to publications in English. DL and ZP did the literature searches, and discrepancies were resolved by discussion with a third author (ZC).

### Eligibility criteria

Eligible articles were considered if they met the following criteria: (1) Participants: women with GDM; (2) Interventions: vitamin supplementation, or mineral supplementation, or vitamin and mineral co-supplementation; (3) Controls: placebo treatment; (4) Outcomes: at least one of the following outcomes was reported, FPG, serum insulin, homeostasis model assessment-insulin resistance (HOMA-IR), homeostasis model of assessment for β cell function (HOMA-B), high-sensitivity C-reactive protein (hs-CRP), total antioxidant capacity (TAC), glutathione (GSH), and malondialdehyde (MDA); (5) Studies: randomized controlled trials (RCTs). Trials were excluded if patients require substitute treatments (such as insulin and metformin et al.), or treatment was medications other than vitamins, minerals or placebo. We also excluded trials without accessible data or full text, or carried out after delivery.

### Data extraction

Data extraction and analysis from included studies were performed by two authors independently (DL and ZP), and conflicts were resolved by a third author (YY). The following information was extracted: first author, publication year, agent, number of participants, dosage, follow-up duration, trial registration number, study location, clinical phase, and outcomes of interest.

### Study quality and risk of Bias assessment

The Cochrane Collaboration’s risk of bias tool was used to assess the risk of bias, including the following items: random sequence generation, allocation concealment, blinding of participants and personnel, blinding of outcome assessment, incomplete outcome data, selective reporting, and other sources of bias. The risk of bias was judged as low, high, or unclear, according to the criteria of Cochrane Handbook.

### Publication Bias assessment

Funnel plot and Egger’s test were used to assess the publication bias and tested for statistical significance. *P* value less than 0.1 was considered significant.

### Statistical analyses

Results were presented as mean difference (MD) with 95% confidence interval (CI). Heterogeneity among studies was estimated by I-squared (*I*^*2*^) tests. *P* value < 0.1 and *I*^*2*^ > 50% indicated statistical heterogeneity, and random-effects models were used in meta-analysis; otherwise, fixed-effects models were used. Sensitivity analyses were also performed to examine the effect of each trial on the overall analysis. All statistical analyses were done with Review manager 5.3 and Stata 12.0.

## Results

### Included studies and baseline characteristics

**Fig. 1:**
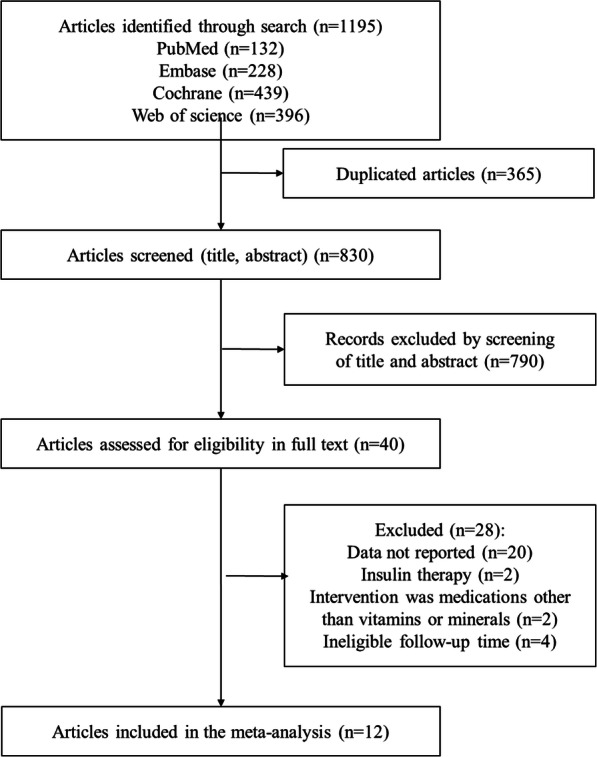
PRISMA flow diagram of study selection process

Our initial search found 1195 potentially relevant articles. After screening, 12 studies were included for meta-analysis [18, 20, 23, 24, 26–33]. The details of study identification and selection were shown in Fig. 1. Table 1 lists key characteristics of the RCTs. Trials included were published between 2013 and 2019. Those 12 selected studies included 698 randomized participants, and all of them were conducted in Iran. Patients included were in the age range of 18–40, and all reported GDM screening was conducted between 24 weeks and 28 weeks gestation. All trials gave vitamin and mineral supplementation orally. Types, doses, dose regimens, and duration of vitamin and mineral supplementation were as follows: magnesium (100–250 mg), zinc (4–233 mg), selenium (200 μg), calcium (400–1000 mg), and vitamin E (400 IU) every day for 6 weeks, vitamin D (200–50,000 IU) every day or every 2 or 3 weeks for 6 weeks. Magnesium, zinc, selenium, calcium, vitamin D or E was given separately or in different combinations: magnesium and vitamin E; zinc and vitamin E; calcium and vitamin D; magnesium, zinc, calcium and vitamin D. All trials used placebo as control intervention.

**Table 1:** Characteristics of the RCTs (randomized controlled trials) included in the meta-analysis

First author, year	agent	Sample size	Dose, frequency	Time, weeks	ClinicalTrials.gov	Country	Phase
Jamilian 2019 [26]	Magnesium-zinc-calcium-vitamin D	60	100 mg magnesium, 4 mg zinc, 400 mg calcium plus 200 IU vitamin D supplements twice a day	6	IRCT201704225623N109	Iran	3
ostadmohammadi 2019 [27]	zinc and vitamin E	60	233 mg/day zinc gluconate plus 400 IU/day vitamin E supplements	6	IRCT20170513033941N26	Iran	3
maktabi 2018 [28]	magnesium and vitamin E	60	250 mg/day magnesium oxide plus 400 IU/day vitamin E supplements	6	IRCT20170513033941N24	Iran	3
karamali 2018 [29]	Magnesium-zinc-calcium-vitamin D	60	100 mg magnesium, 4 mg zinc, 400 mg calcium plus 200 IU vitamin D supplements twice a day	6	–	Iran	–
jamilian 2017 [30]	Magnesium	40	250 mg/day of magnesium supplements	6	IRCT201704235623N111	Iran	3
razavi 2017 [23]	vitamin D	60	50,000 IU vitamin D every 2 weeks	6	IRCT201701305623N106	Iran	3
karamali 2016 [31]	zinc	50	233 mg zinc gluconate (containing 30 mg zinc)	6	IRCT201503055623N42	Iran	–
karamali 2015 [32]	zinc	58	233 mg zinc gluconate (containing 30 mg zinc) per day	6	IRCT201408295623N26	Iran	3
asemi 2015–1 [20]	selenium	70	200 μg selenium supplements/day	6	IRCT201403175623N18	Iran	2
asemi 2015–2 [33]	magnesium	70	250 mg magnesium supplements/day	6	IRCT201503055623N39	Iran	2
asemi 2014 [24]	Calcium plus vitamin D	56	1000 mg calcium carbonate per day plus 50,000 IU vitamin D3 every 3 weeks	6	IRCT201311205623N11	Iran	2
asemi 2013 [18]	Vitamin D	54	50,000 IU vitamin D3 every 3 weeks	6	IRCT201305115623N7	Iran	2

### Risk of bias of individual studies

**Fig. 2:**
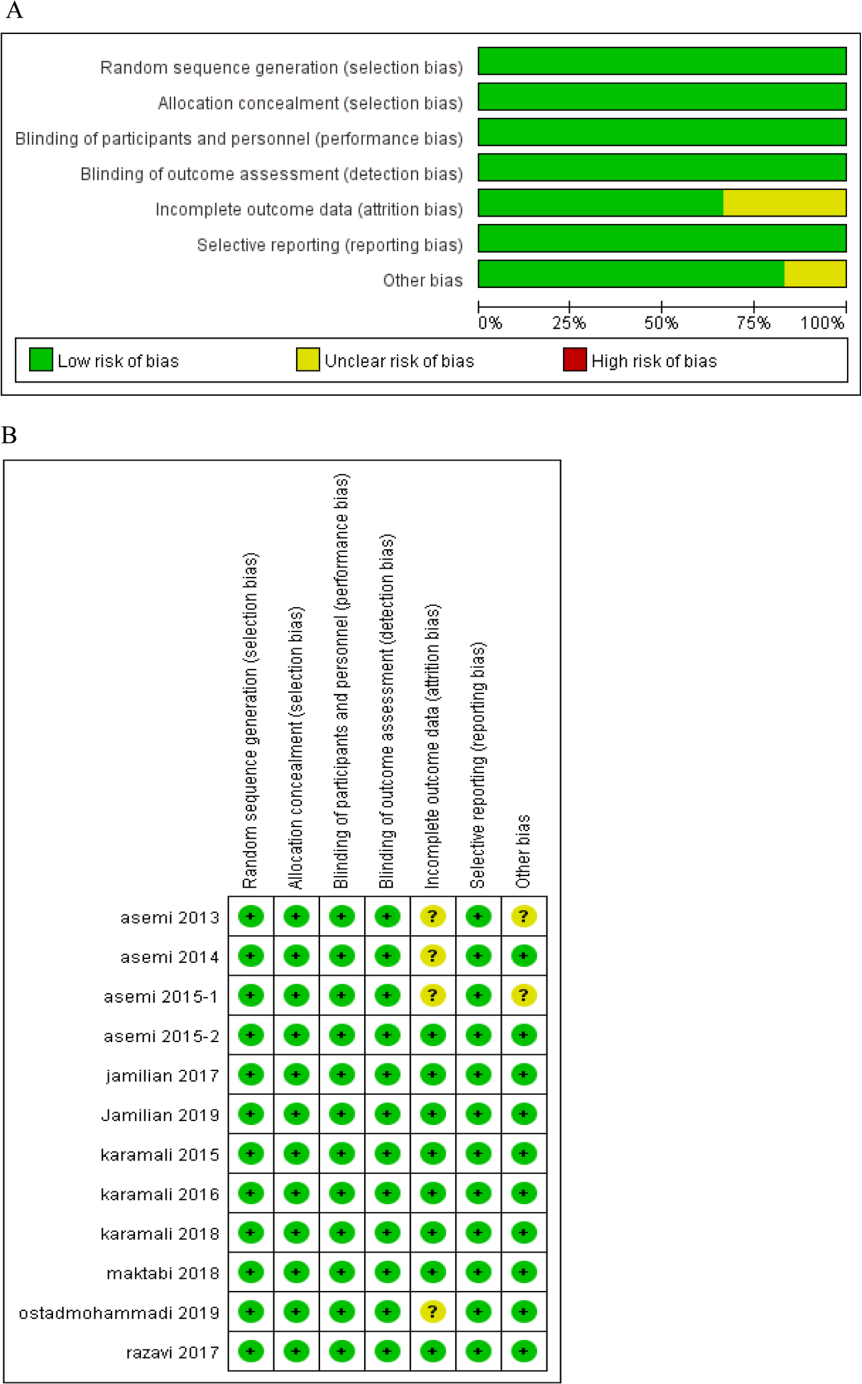
Risk of bias **a** and risk of bias summary **b**. In Fig. 2**a**, green represents a low risk of bias, while yellow represents unclear risk of bias. In Fig. 2**b**, + represents a low risk of bias, while ? represents unclear risk of bias

The quality of the included trails was assessed according to the criteria of Cochrane Handbook, and the results of risk of bias and risk of bias summary are summarized in Fig. 2. Among the 12 trials, 8 were judged to be at low risk of bias and 4 as being at unclear risk of bias. Unclear risks were related to attrition bias and other bias.

### Meta-analysis

#### The effects of vitamin and mineral supplementation on glycemic control

**Fig. 3:**
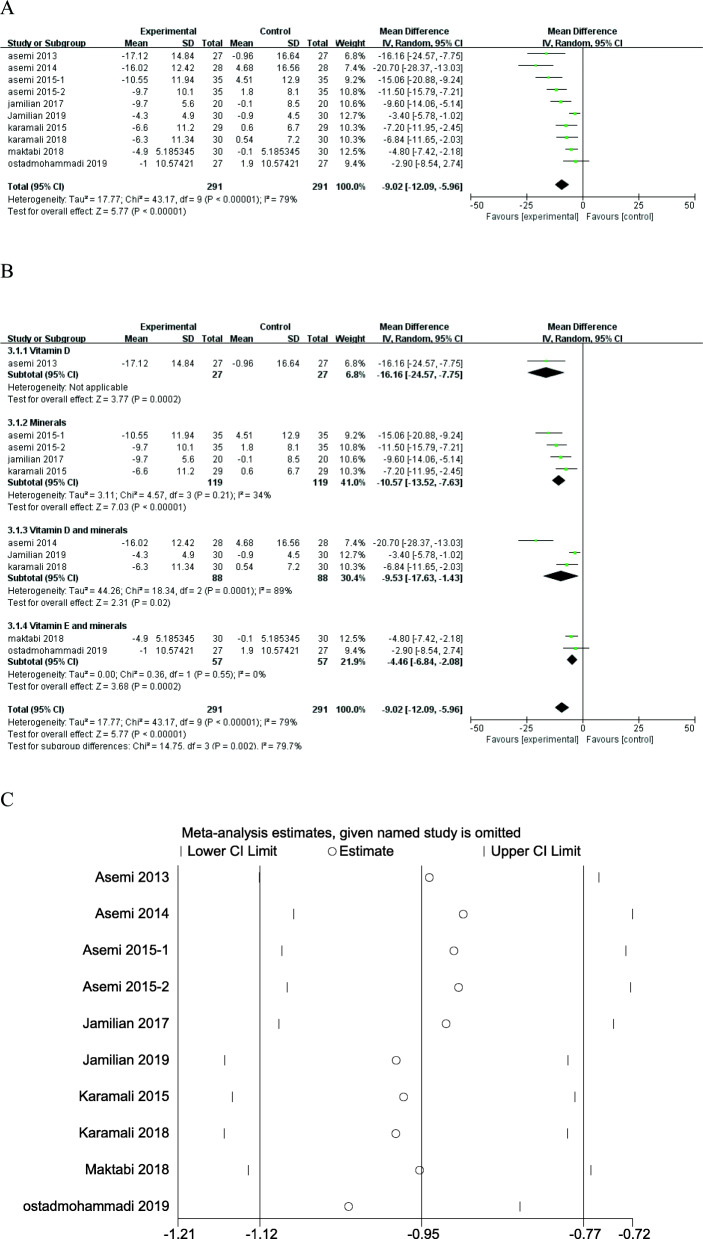
Forest plot of pooled MD and sensitivity analysis in FPG of studies included. **a** Meta-analysis of the effects of vitamin and mineral supplementation on FPG in women with GDM (random-effects model); **b** The forest plot of FPG in subgroup analysis defined by the type of interventions (random-effects model); **c** Sensitivity analysis in FPG of studies included. Mean difference, MD; Fasting plasma glucose, FPG; gestational diabetes mellitus, GDM

Ten RCTs examined the effects of vitamin and mineral supplementation on FPG level among patients with GDM. The pooled results indicated that FPG level decreased significantly following vitamin and mineral supplementation (MD = - 9.02; 95% CI: - 12.09, - 5.96; *P* < 0.00001) (Fig. 3a). There was significant heterogeneity among studies (*I*^*2*^ = 79%; *P* < 0.00001). Subgroup analysis was performed according to the type of interventions, and there are four subgroups including vitamin D and minerals, vitamin E and minerals, vitamin D, and minerals. Taking individual vitamin and mineral supplementation (50,000 IU vitamin D every 3 weeks, 200 μg/d selenium, 250 mg /d magnesium or 233 mg/d zinc) could significantly decrease the FPG level in GDM patients (Fig. 3b). Similar results were found in combined vitamin and mineral supplementation: 400 IU/d vitamin E plus either 250 mg/d magnesium or 233 mg/d zinc (MD = - 4.46; 95% CI: - 6.84, - 2.08; *P* = 0.0002) (Fig. 3b), 100 mg magnesium, 4 mg zinc, 400 mg calcium plus 200 IU vitamin D supplementation twice a day, or 1000 mg calcium/d plus 50,000 IU vitamin D3 every 3 weeks (MD = - 9.53; 95% CI: - 17.63, - 1.43; *P* = 0.02) (Fig. 3b). Sensitivity analysis of FPG showed that the removal of any given named study, the pooled results were largely unchanged (Fig. 3c).

**Fig. 4:**
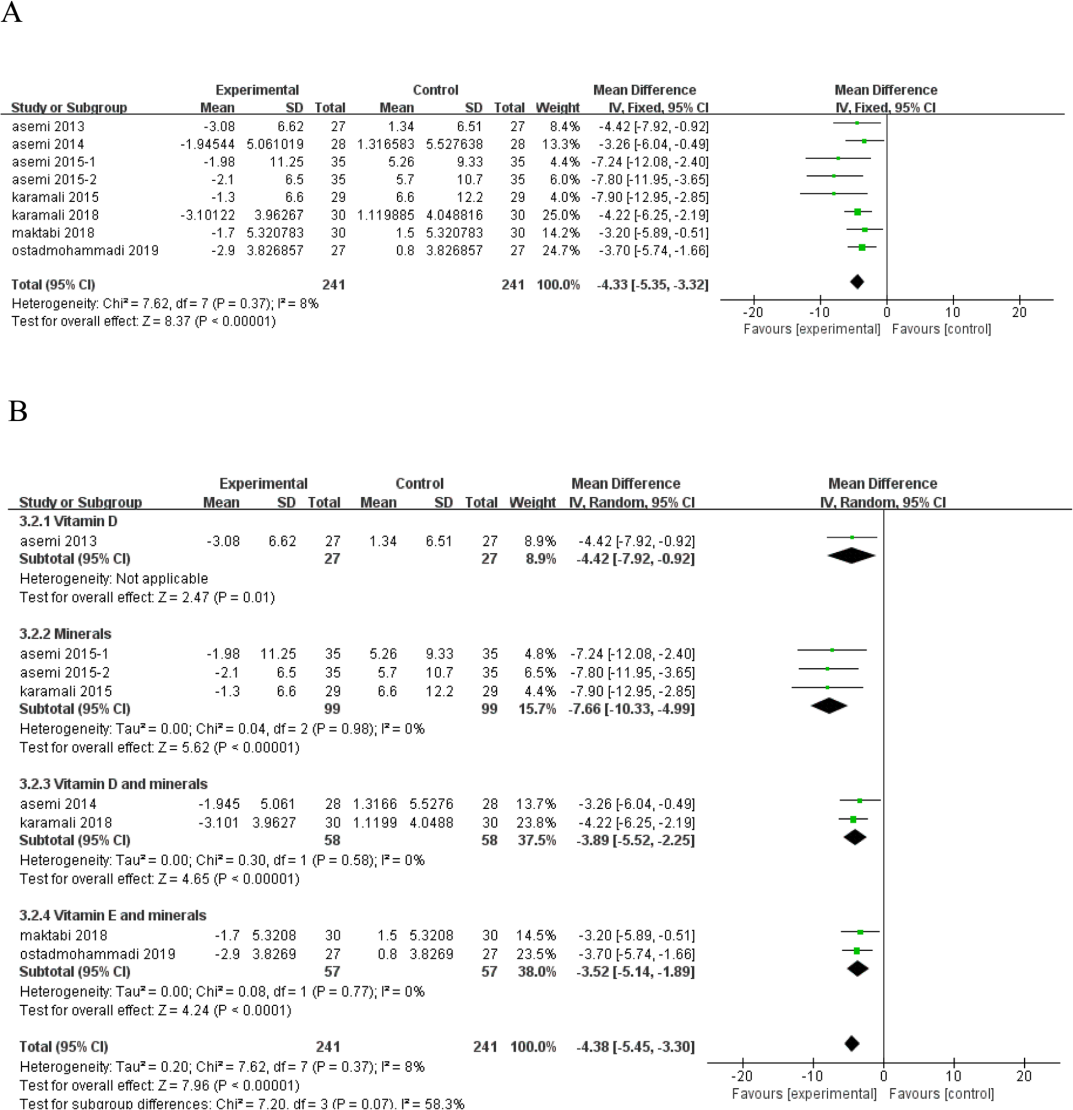
Forest plot of pooled MD in serum insulin of studies included. **a** Meta-analysis of the effects of vitamin and mineral supplementation on serum insulin in women with GDM (fixed-effects model); **b** The forest plot of serum insulin in subgroup analysis defined by the type of interventions (random-effects model). Mean difference, MD; gestational diabetes mellitus, GDM

Vitamin and mineral supplementation was found to significantly reduce serum insulin levels (*n* = 8; MD = - 4.33; 95% CI: - 5.35, - 3.32; *P* < 0.00001) (Fig. 4a). Subgroup analysis according to type of interventions removed the heterogeneity between studies in serum insulin (Fig. 4b).

**Fig. 5:**
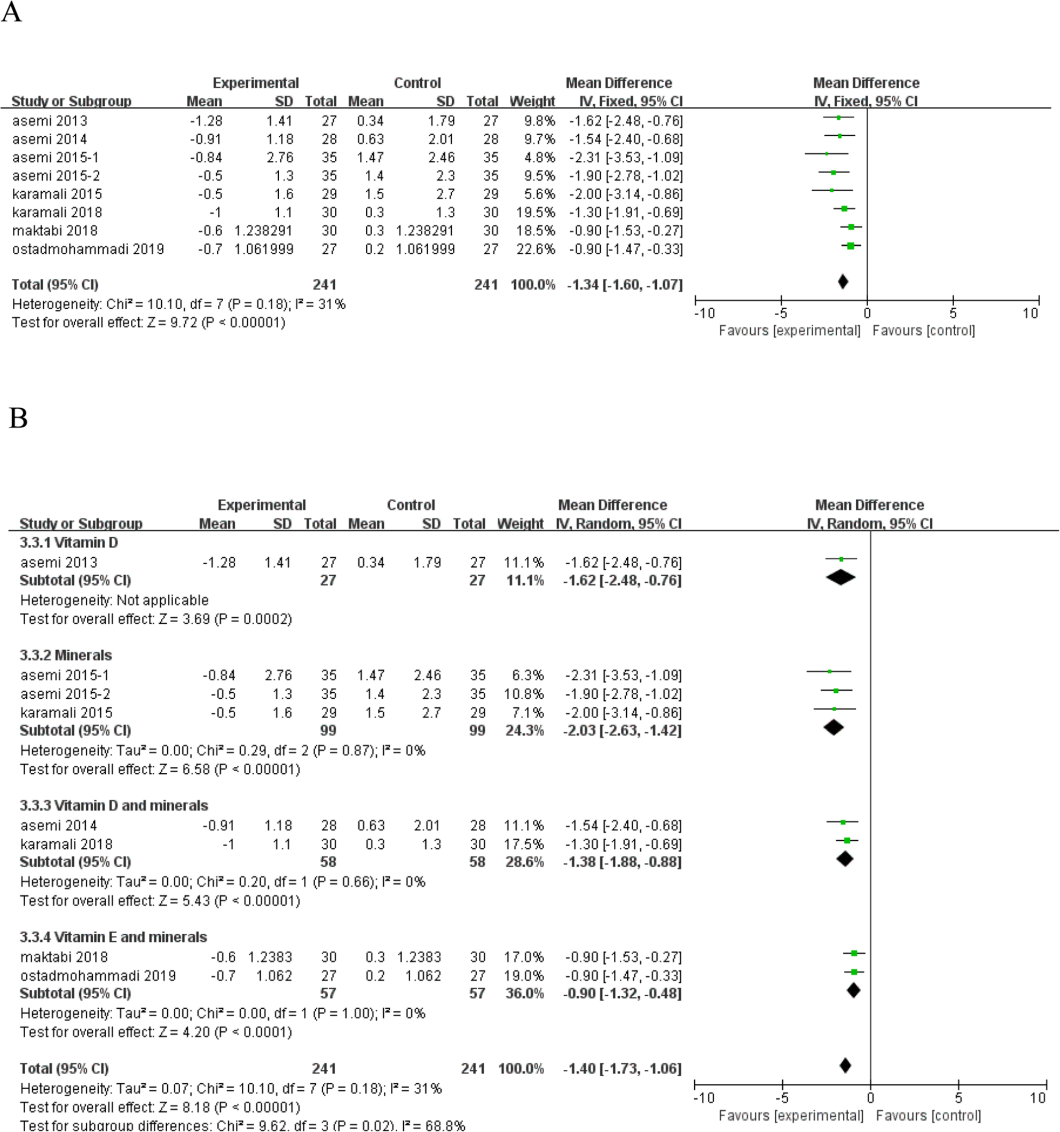
Forest plot of pooled MD in HOMA-IR of studies included. **a** Meta-analysis of the effects of vitamin and mineral supplementation on HOMA-IR in women with GDM (fixed-effects model); **b** The forest plot of HOMA-IR in subgroup analysis defined by the type of interventions (random-effects model). Mean difference, MD; Homeostasis model assessment-insulin resistance, HOMA-IR; gestational diabetes mellitus, GDM

HOMA-IR level decreased significantly following vitamin and mineral supplementation (*n* = 8; MD = - 1.34; 95% CI: - 1.60, - 1.07; *P* < 0.00001) (Fig. 5a), with low heterogeneity among studies. Subgroup analysis according to type of interventions removed the heterogeneity between studies in HOMA-IR (Fig. 5b), and the pooled results were largely unchanged.

**Fig. 6:**
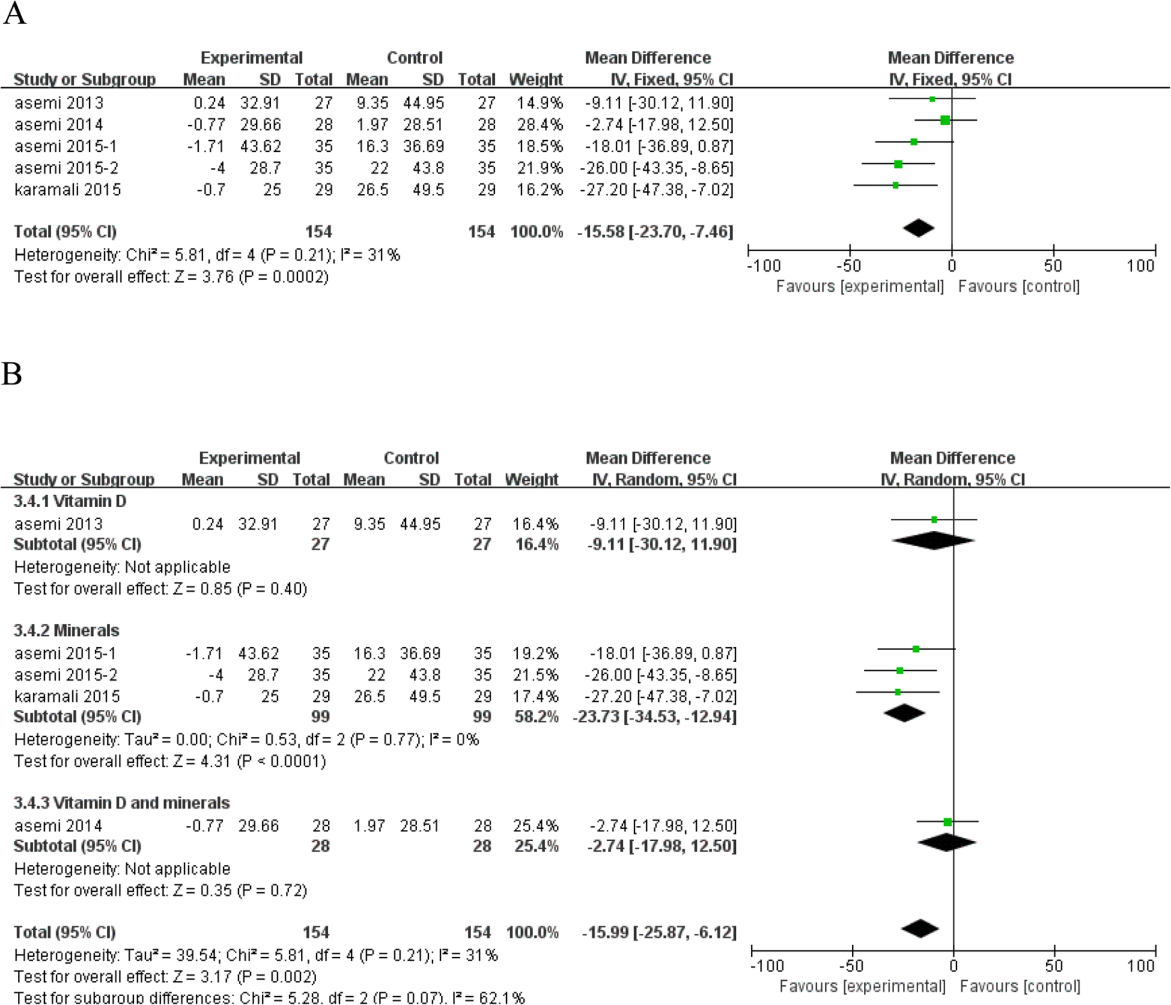
Forest plot of pooled MD in HOMA-B of studies included. **a** Meta-analysis of the effects of vitamin and mineral supplementation on HOMA-B in women with GDM (fixed-effects model); **b** The forest plot of HOMA-B in subgroup analysis defined by the type of interventions (random-effects model). Mean difference, MD; Homeostasis model of assessment for β cell function, HOMA-B; gestational diabetes mellitus, GDM

Vitamin and mineral supplementation significantly reduced the HOMA-B levels (*n* = 5; MD = - 15.58; 95% CI: - 23.70, - 7.46; *P* = 0.0002) (Fig. 6a). Subgroup analysis showed that mineral supplementation, such as 200 μg selenium/d, 250 mg magnesium/d, or 233 mg zinc gluconate/d, could significantly reduce the HOMA-B levels (MD = - 23.73; 95% CI: - 34.53, - 12.94; *P* < 0.0001), while vitamin D, vitamin D and calcium co-supplementation have no significant effect on HOMA-B levels (Fig. 6b).

#### The effects of vitamin and mineral supplementation on inflammatory markers

**Fig. 7:**
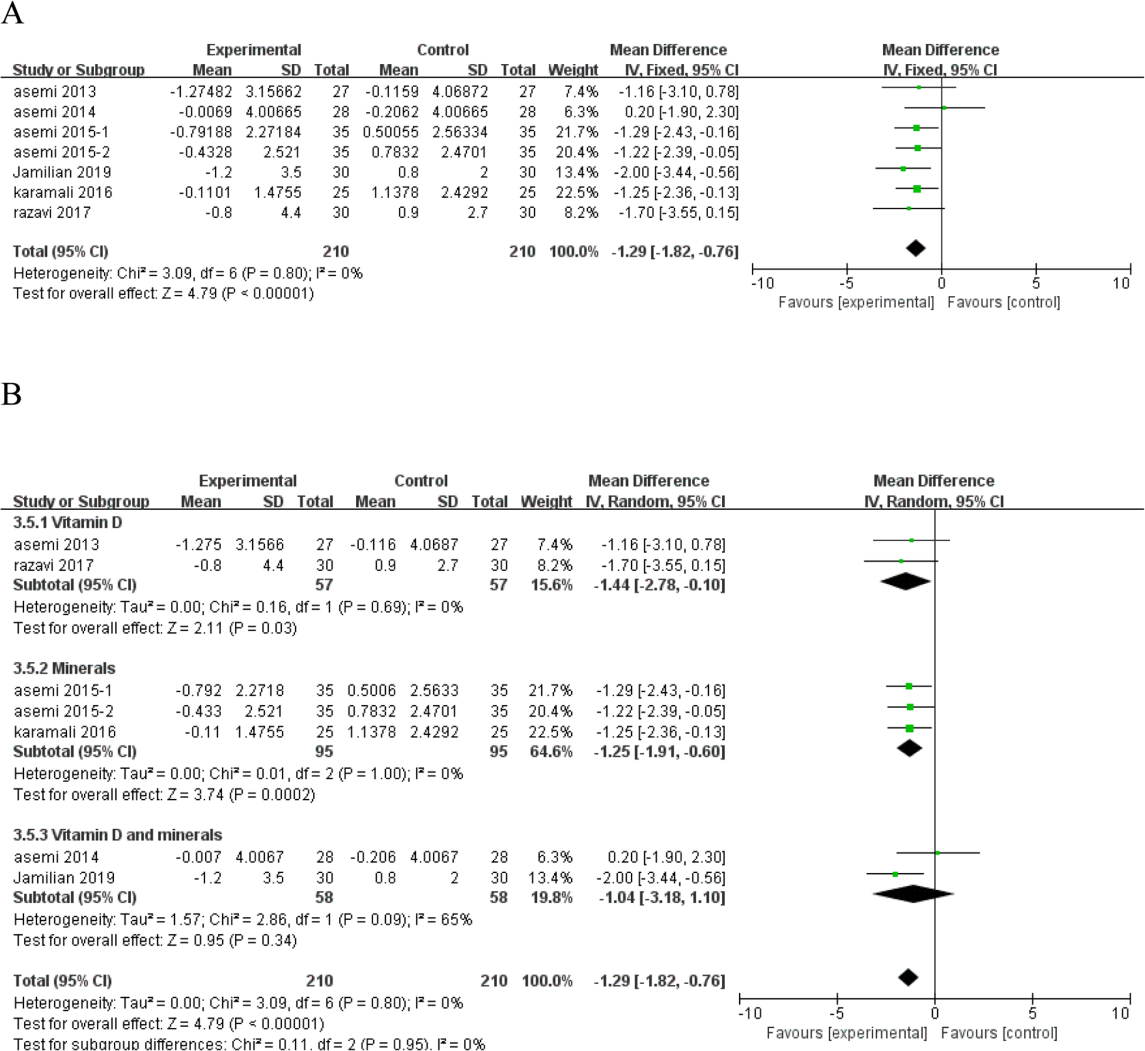
Forest plot of pooled MD in hs-CRP of studies included. **a** Meta-analysis of the effects of vitamin and mineral supplementation on hs-CRP in women with GDM (fixed-effects model); **b** The forest plot of hs-CRP in subgroup analysis defined by the type of interventions (random-effects model). Mean difference, MD; High-sensitivity C-reactive protein, hs-CRP; gestational diabetes mellitus, GDM

Figure 7a shows the forest plots for inflammatory markers hs-CRP. The results showed that vitamin and mineral supplementation significantly reduced the hs-CRP level (*n* = 7; MD = - 1.29; 95% CI: - 1.82, - 0.76; *P* < 0.00001), with no heterogeneity among the studies (*I*^*2*^ = 0%, heterogeneity *P* = 0.80). However, subgroup analysis according to the type of interventions showed that vitamin D and minerals co-supplementation has no significant effect on hs-CRP levels with moderate heterogeneity between studies (Fig. 7b).

#### The effects of vitamin and mineral supplementation on biomarkers of oxidative stress

**Fig. 8:**
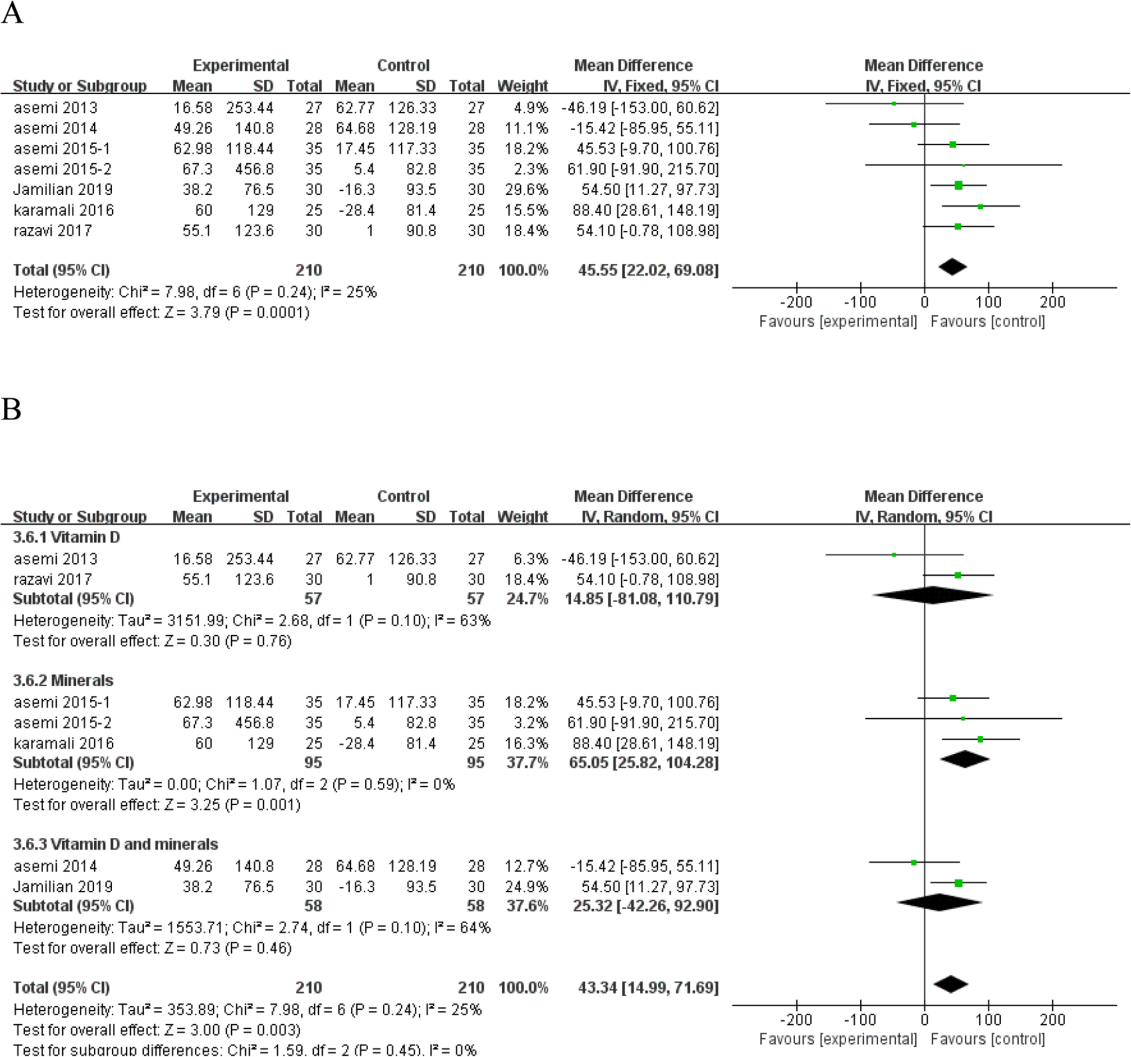
Forest plot of pooled MD in TAC of studies included. **a** Meta-analysis of the effects of vitamin and mineral supplementation on TAC in women with GDM (fixed-effects model); **b** The forest plot of TAC in subgroup analysis defined by the type of interventions (random-effects model). Mean difference, MD; Total antioxidant capacity, TAC; gestational diabetes mellitus, GDM

TAC is considered to be an indicator of the oxygen radical absorbance capacity. Vitamin D, minerals and vitamin D plus mineral co-supplementation significantly increased TAC level compared with placebo (MD = 45.55; 95% CI: 22.02, 69.08; *P* = 0.0001) (Fig. 8a), with low heterogeneity among studies (*I*^*2*^ = 25%, heterogeneity *P* = 0.24). Subgroup analysis of TAC according to the type of interventions showed that mineral subgroup, such as selenium, magnesium, or zinc, could significantly increase TAC level, while vitamin D alone or vitamin D and mineral co-supplementation has no significant effect on TAC levels, with moderate heterogeneity among studies (Fig. 8b).

**Fig. 9:**
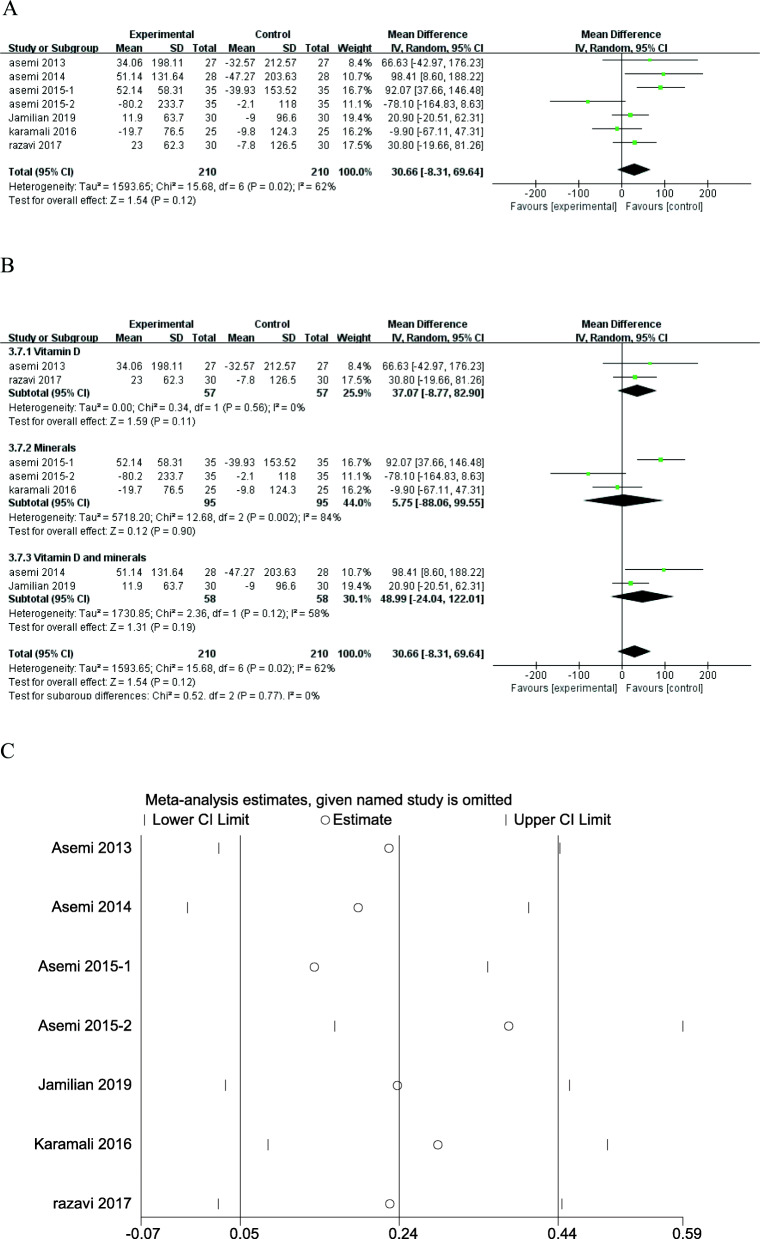
Forest plot of pooled MD and sensitivity analysis in GSH of studies included. **a** Meta-analysis of the effects of vitamin and mineral supplementation on GSH in women with GDM (random-effects model); **b** The forest plot of GSH in subgroup analysis defined by the type of interventions (random-effects model); **c** Sensitivity analysis in GSH of studies included. Mean difference, MD; Glutathione, GSH; gestational diabetes mellitus, GDM

GSH is an endogenous antioxidant, but vitamin and mineral supplementation did not significantly affect GSH level (MD = 30.66; 95% CI: - 8.31, 69.64; *P* = 0.12) (Fig. 9a), and there was moderate heterogeneity among studies (*I*^*2*^ = 62%; *P* = 0.02). Subgroup analysis (Fig. 9b) and sensitivity analysis (Fig. 9c) of GSH according to the type of interventions didn’t change the results.

**Fig. 10:**
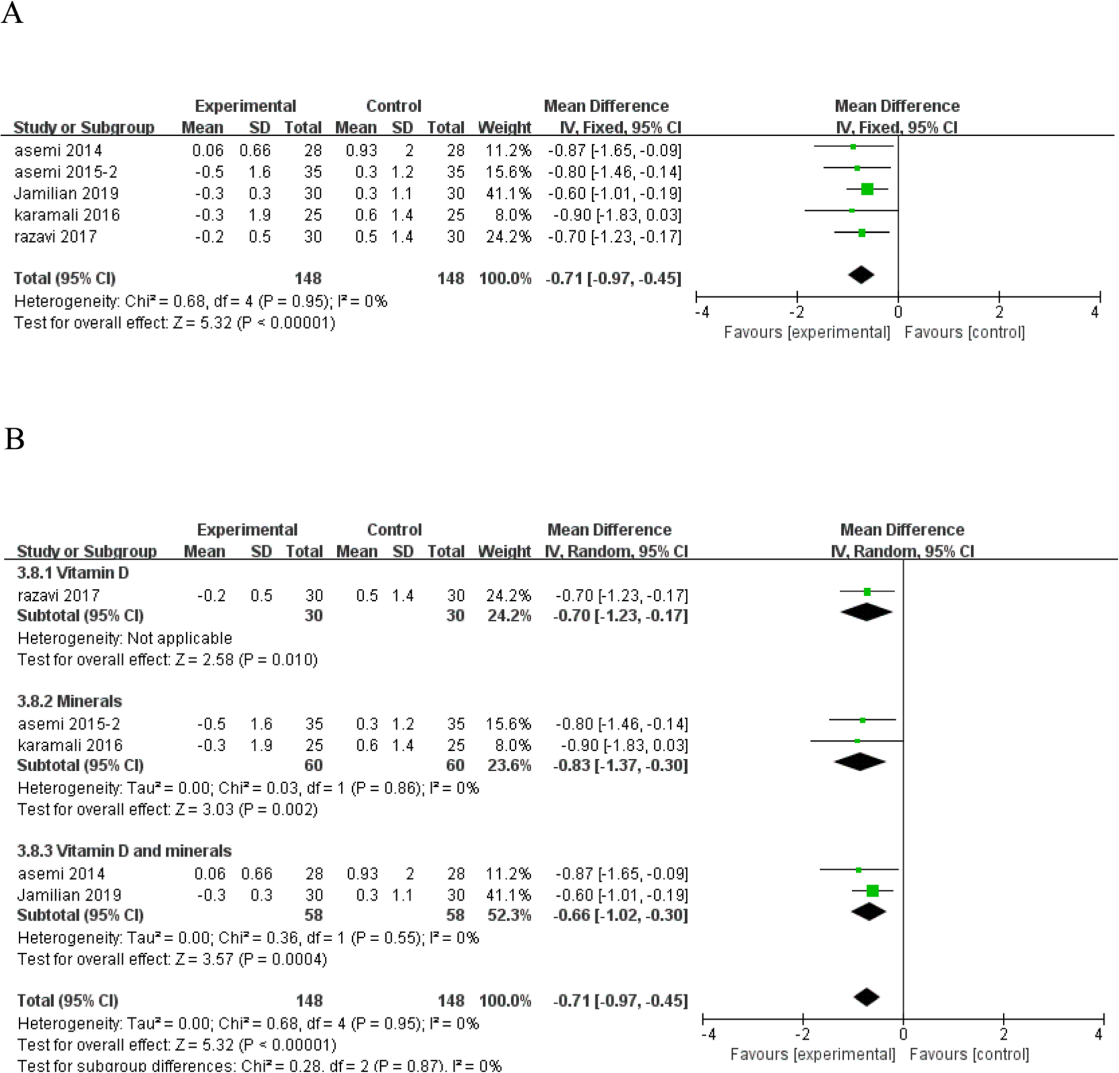
Forest plot of pooled MD in MDA of studies included. **a** Meta-analysis of the effects of vitamin and mineral supplementation on MDA in women with GDM (fixed-effects model); **b** The forest plot of MDA in subgroup analysis defined by the type of interventions (random-effects model). Mean difference, MD; Malondialdehyde, MDA; gestational diabetes mellitus, GDM

Vitamin and mineral supplementation significantly reduced MDA (MD = - 0.71; 95% CI: - 0.97, - 0.45; *P* < 0.00001) (Fig. 10a), an indicator of lipid peroxide formation, and no heterogeneity was found among the studies (*I*^*2*^ = 0%, heterogeneity *P* = 0.95). In subgroup analysis according to the type of interventions, the effects of vitamin and mineral supplementation on MDA levels stay the same (Fig. 10b).

### Publication bias

No significant publication bias was found for the effect of vitamin and mineral supplementation on all eight outcomes (Fig. S1).

## Discussion

In this meta-analysis, we pooled the results of high-quality RCTs in women with GDM to assess the effects of vitamin and mineral supplementation vs placebo on glycemic control, biomarkers of inflammation and oxidative stress. Due to the close metabolism regulation between vitamins and minerals and data on the effects of vitamin and mineral co-administration on glucose regulation, inflammation and oxidative stress markers in women with GDM are scarce, we specifically included vitamin and mineral co-administration trails in this meta-analysis. And in order to have more reliable results and decrease the heterogeneity between different studies, we didn’t include trials if patients require substitute treatments (such as insulin and metformin et al.) or carried out after delivery, since both substitute treatments and delivery can affect glycemic status. Our findings demonstrated that vitamin and mineral supplementation, magnesium, zinc, selenium, calcium, vitamin D and E (alone or in combination), significantly improved glycemic control, attenuated inflammation and oxidative stress in women with GDM through decreasing serum FPG, insulin, HOMA-IR, HOMA-B, hs-CRP and MDA levels, and increasing TAC levels.

### Vitamin and mineral supplementation improved glycemic control in GDM and related mechanism

Findings from this meta-analysis demonstrated that vitamin and mineral supplementation had a significant effect on serum insulin and blood glucose parameters. Subgroup analysis of the interventions showed that single mineral or vitamin D, or combined vitamin D/E and mineral supplementation all worked well on glycemic control. We found that vitamin D, magnesium and zinc has the potential role to promote glycemic control in diabetes patients. The dosages of vitamin D might be a reason of high heterogeneity of FPG in vitamin D and minerals group, 50,000 IU vitamin D every 3 weeks plus 1000 mg calcium per day worked better than 200 IU vitamin D supplementation plus minerals (100 mg magnesium, 4 mg zinc and 400 mg calcium) twice a day. Although different doses of vitamin D supplementation, ranging from 200 to 4000 IU daily for 12.5 days, had no effect on FPG level in women with GDM, higher doses of vitamin D significantly improved insulin resistance [5]. Vitamin D can activate the transcription of insulin receptor gene to promote glucose oxidation through the phosphoinositide 3-kinase (PI3-K) pathway [34]. Vitamin E plays an important role in regulating glucose metabolism through improve insulin action by upregulation GSH/GSSG ratio and magnesium concentration [13]. Magnesium can regulate glucose and insulin metabolism by affecting the tyrosine kinase activity of insulin receptor, and help transport glucose into the cells via glucose transporter protein activity 4 (GLUT4) [35]. Zinc can regulate insulin signaling through PI3-K/Akt pathway and help transport GLUT4 to the cell membrane [14].

### Vitamin and mineral supplementation relieves inflammation and possible mechanisms in GDM

Pregnant women with obesity or GDM have increased maternal inflammation and elevated hs-CRP compared to normal pregnant women, and suppression the inflammation helps improving pregnancy outcomes and maternal complications. Our meta-analysis showed that the supplementation of vitamin and mineral could significantly reduce the serum hs-CRP level in GDM patients. Subgroup analysis of treatments demonstrated that mineral supplementation, including zinc, magnesium, and selenium, was more significant than vitamin D and mineral supplementation in reducing hs-CRP level. Vitamin D, zinc and selenium can decrease the concentration of inflammatory cytokines through down-regulation of nuclear transcription factor κB (NF-κB), a key regulator of genes involved in inflammation [14, 36]. Magnesium may decrease inflammatory response via regulation the intracellular calcium concentration and inhibition the NF-κB signaling pathway [37, 38].

### Underlying mechanisms of vitamin and mineral supplementation on oxidative stress in GDM

Oxidative stress plays a key role in the development of diabetes mellitus [39]. Many kinds of vitamins and minerals have antioxidant activity and may have beneficial role in diabetes. Our meta-analysis showed a benefit effect of vitamin and mineral supplementation, including vitamin D, Calcium plus vitamin D, zinc and magnesium, on alleviating oxidative stress status in GDM patients through improving TAC levels, and reducing MDA levels. Vitamin D can increase GSH formation by upregulation of glutamate cysteine ligase (GCLC) and glutathione reductase (GR), which recycles oxidized GSSG to GSH to scavenge excessive ROS [40]. Magnesium can influence the activities of mitochondrial electron transport chain and ROS production [41]. Zinc is essential for the activity of superoxide dismutase (SOD), which plays an important role in redox balance, and may play antioxidant effect by decreasing ROS production [14].

There are interactions between different vitamins and minerals. Zinc can regulate the release of calcium by acting on zinc-sensing receptor [42], vitamin D affects insulin secretion by regulating plasma calcium [43], vitamin E can improve glucose disposal through the upregulation of magnesium concentration [13], magnesium is involved in the synthesis, transport and activation of vitamin D [44]. Because of the possible interactions between different vitamins and minerals, further studies are necessary to assess whether the combination of vitamins and minerals is more effective than a single vitamin or mineral on GDM.

### Limitations of our study

There are several limitations in our meta-analysis. First, the diversity of vitamin and mineral types and combinations make it challenging to perform subgroup analysis for all treatments. Second, all included trails were from Iran, and there was a lack of data from other countries. Finally, since GDM usually diagnosed between 24- and 28-week gestation, we didn’t include studies last more than 3 months to see the effect on postpartum outcomes.

## Conclusion

In conclusion, our meta-analysis suggests a beneficial role of single or vitamin and mineral co-supplementation on biomarkers of glycemic control, inflammation and oxidative stress in women with GDM. In the future, more large-scale and longer duration studies of vitamin and mineral supplementation, especially vitamin and mineral co-supplementation, are needed to demonstrate their effect on glycemic control, anti-inflammatory and antioxidant effects in women with GDM.

## Supplementary Information

**Additional file 1:** Full search strategy in database. Search strategy of Pubmed (A), Embase (B), Web of Sciense (C) and Cochrane Library (D).

**Additional file 2: Figure S1** Funnel plot for publication bias test of included studies for FPG (A), serum insulin (B), HOMA-IR (C), HOMA-B (D), hs-CRP (E), TAC (F), GSH (G), and MDA (H). Fasting plasma glucose, FPG; Homeostasis model assessment-insulin resistance, HOMA-IR; Homeostasis model of assessment for β cell function, HOMA-B; High-sensitivity C-reactive protein, hs-CRP; Total antioxidant capacity, TAC; Glutathione, GSH; Malondialdehyde, MDA

## Data Availability

The datasets used and/or analyzed during the current study are available from the corresponding author on reasonable request.

## Abbreviations

GDM: Gestational diabetes mellitus
T2D: Type 2 diabetes
CVD: Cardiovascular disease
IRS: Insulin resistance syndrome
FPG: Fasting plasma glucose
FBG: Fasting blood glucose
PRISMA: Preferred Reporting Items for Systematic Reviews and Meta-Analyses
MeSH: Medical Subject Headings
HOMA-IR: Homeostasis model assessment-insulin resistance
HOMA-B: Homeostasis model of assessment for β cell function
hs-CRP: High-sensitivity C-reactive protein
TAC: Total antioxidant capacity
GSH: Glutathione
MDA: Malondialdehyde
RCTs: Randomized controlled trials
MD: Mean difference
CI: Confidence interval
I^2^: I-squared
PI3-K: Phosphoinositide 3-kinase
GLUT4: Glucose transporter protein activity 4
NF-κB: Nuclear transcription factor κB
GCLC: Glutamate cysteine ligase
GR: Glutathione reductase
SOD: Superoxide dismutase
