# Supplementary material for: The effects of vitamin and mineral supplementation on women with gestational diabetes mellitus
Source: BMC Endocr Disord. 2021 May 24;21:106. doi: 10.1186/s12902-021-00712-x (PMC8145819; doi:10.1186/s12902-021-00712-x)
Supplement: Supplementary file 1 — Additional file 1: Full search strategy in database. Search strategy of Pubmed (A), Embase (B), Web of Sciense (C) and Cochrane Library (D). [file 12902_2021_712_MOESM1_ESM.docx]

**Additional file 1**: Full search strategy in database. Search strategy of Pubmed (A), Embase (B), Web of Sciense (C) and Cochrane Library (D).

A

#1 "diabetes, gestational"[MeSH Terms]

#2 "pregnancy diabetes mellitus"[Title/Abstract] OR "gestational diabetes mellitus"[Title/Abstract] OR "gestational diabetes"[Title/Abstract] OR "GDM"[Title/Abstract]

#3 #1 OR #2

#4 "micronutrients"[MeSH Terms]

#5 "micronutrient*"[Title/Abstract] OR "vitamin*"[Title/Abstract] OR "mineral*"[Title/Abstract] OR "iron"[Title/Abstract] OR "calcium"[Title/Abstract] OR "magnesium"[Title/Abstract] OR "zinc"[Title/Abstract] OR "selenium"[Title/Abstract]

#6 #4 OR #5

#7 "glycaemic control"[Title/Abstract] OR "glucose"[Title/Abstract] OR "insulin"[Title/Abstract] OR "oxidative stress*"[Title/Abstract] OR "antioxidant"[Title/Abstract] OR "inflammation"[Title/Abstract] OR "inflammatory"[Title/Abstract]

#8 "randomized controlled trial"[Publication Type] OR "random*"[Title/Abstract] OR "controlled"[Title/Abstract] OR "trial*"[Title/Abstract] OR "placebo"[Title/Abstract]

#9 #3 AND #6 AND #7 AND #8

B

#1 'pregnancy diabetes mellitus'/exp

#2 'pregnancy diabetes mellitus':ti,ab,kw OR 'gestational diabetes mellitus':ti,ab,kw OR 'gestational diabetes':ti,ab,kw OR gdm:ti,ab,kw

#3 #1 OR #2

#4 'vitamin'/exp

#5 'mineral'/exp

#6 vitamin*:ti,ab,kw OR mineral*:ti,ab,kw OR iron:ti,ab,kw OR calcium:ti,ab,kw OR magnesium:ti,ab,kw OR zinc:ti,ab,kw OR selenium:ti,ab,kw OR micronutrient*:ti,ab,kw

#7 #4 OR #5 OR #6

#8 'glycaemic control':ti,ab,kw OR glucose:ti,ab,kw OR insulin:ti,ab,kw OR 'oxidative stress*':ti,ab,kw OR antioxidant:ti,ab,kw OR inflammation:ti,ab,kw OR inﬂammatory:ti,ab,kw

#9 'randomized controlled trial':it OR random*:ab,ti OR controlled:ab,ti OR trial*:ab,ti OR placebo:ab,ti

#10 #3 AND #7 AND #8 AND #9

C

#1 TI=(“pregnancy diabetes mellitus” OR “gestational diabetes mellitus” OR “gestational diabetes” OR gdm) OR AB=(“pregnancy diabetes mellitus” OR “gestational diabetes mellitus” OR “gestational diabetes” OR gdm) OR AK=(“pregnancy diabetes mellitus” OR “gestational diabetes mellitus” OR “gestational diabetes” OR gdm)

#2 TI=(vitamin* OR mineral* OR iron OR calcium OR magnesium OR zinc OR selenium OR micronutrient*) OR AB=(vitamin* OR mineral* OR iron OR calcium OR magnesium OR zinc OR selenium OR micronutrient*) OR AK=(vitamin* OR mineral* OR iron OR calcium OR magnesium OR zinc OR selenium OR micronutrient*)

#3 TI=(glycaemic control OR glucose OR insulin OR oxidative stress* OR antioxidant OR inflammation OR inﬂammatory) OR AB=(glycaemic control OR glucose OR insulin OR oxidative stress* OR antioxidant OR inflammation OR inﬂammatory) OR AK=(glycaemic control OR glucose OR insulin OR oxidative stress* OR antioxidant OR inflammation OR inﬂammatory)

#4 TI=(random* OR controlled OR trial* OR placebo) OR AB=(random* OR controlled OR trial* OR placebo) OR AK=(random* OR controlled OR trial* OR placebo)

#5 #1 AND #2 AND #3 AND #4

D

#1 MeSH descriptor: [Diabetes, Gestational] explode all trees

#2 (pregnancy diabetes mellitus):ti,ab,kw OR (gestational diabetes mellitus):ti,ab,kw OR (gestational diabetes):ti,ab,kw OR (GDM):ti,ab,kw (Word variations have been searched)

#3 #1 OR #2

#4 MeSH descriptor: [Micronutrients] explode all trees

#5 (Micronutrient*):ti,ab,kw OR (Vitamin*):ti,ab,kw OR (Mineral*):ti,ab,kw OR (Iron):ti,ab,kw OR (Calcium):ti,ab,kw (Word variations have been searched)

#6 (Magnesium):ti,ab,kw OR (Zinc):ti,ab,kw OR (Selenium):ti,ab,kw (Word variations have been searched)

#7 #4 OR #5 OR #6

#8 (Glycaemic Control):ti,ab,kw OR (Glucose):ti,ab,kw OR (insulin):ti,ab,kw OR (oxidative stress*):ti,ab,kw OR (antioxidant):ti,ab,kw (Word variations have been searched)

#9 (Inflammation):ti,ab,kw OR (inﬂammatory):ti,ab,kw (Word variations have been searched)

#10 #8 OR #9

#11 #3 AND #7 AND #10
