# Supplementary material for: The effects of vitamin and mineral supplementation on women with gestational diabetes mellitus
Source: BMC Endocr Disord. 2021 May 24;21:106. doi: 10.1186/s12902-021-00712-x (PMC8145819; doi:10.1186/s12902-021-00712-x)

**Additional file 2: Fig. S1** Funnel plot for publication bias test of included studies for FPG (A), serum insulin (B), HOMA-IR (C), HOMA-B (D), hs-CRP (E), TAC (F), GSH (G), and MDA (H). Fasting plasma glucose, FPG; Homeostasis model assessment-insulin resistance, HOMA-IR; Homeostasis model of assessment for β cell function, HOMA-B; High-sensitivity C-reactive protein, hs-CRP; Total antioxidant capacity, TAC; Glutathione, GSH; Malondialdehyde, MDA.


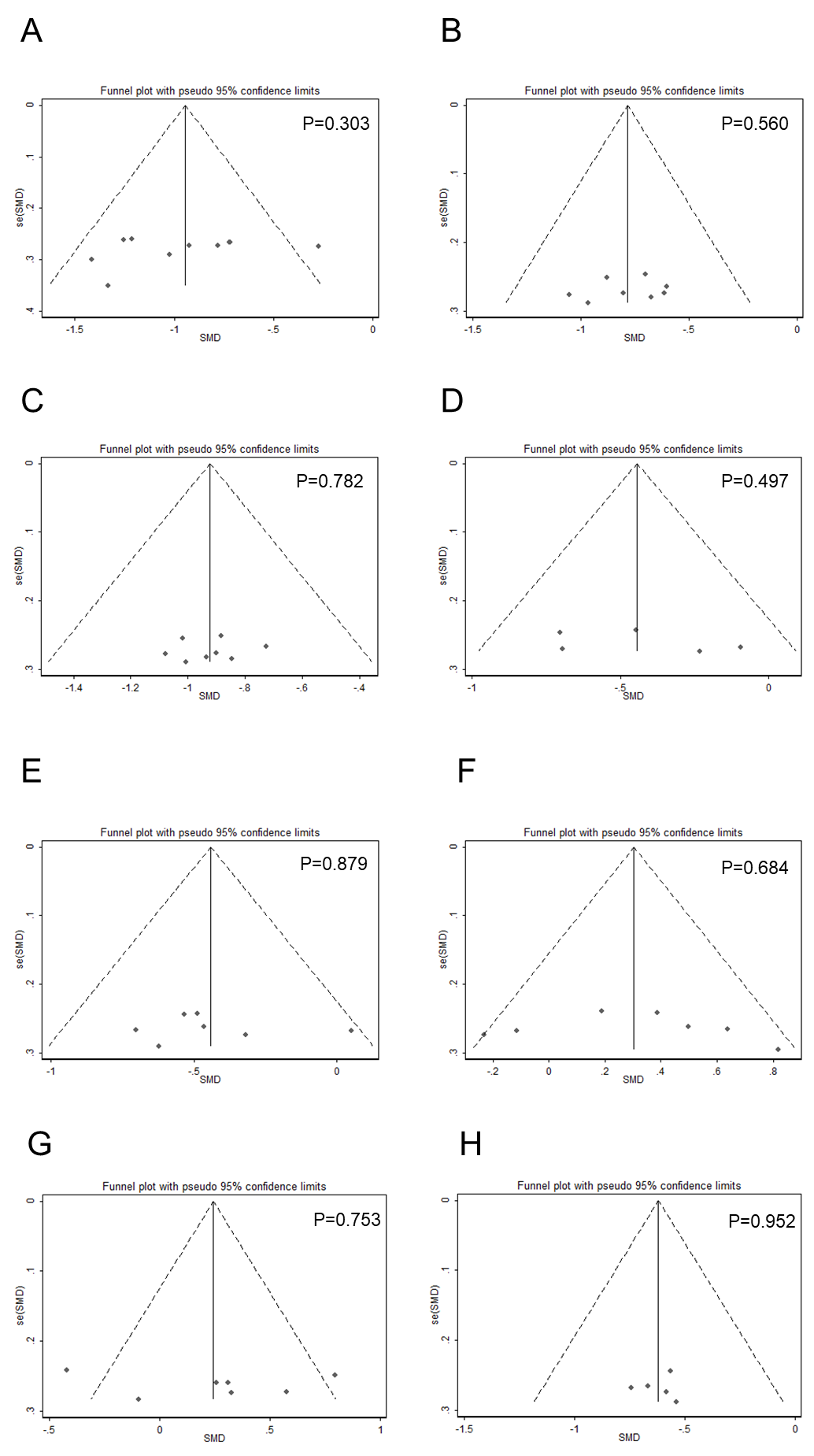

Supplement: Supplementary file 2 — Additional file 2: Figure S1 Funnel plot for publication bias test of included studies for FPG (A), serum insulin (B), HOMA-IR (C), HOMA-B (D), hs-CRP (E), TAC (F), GSH (G), and MDA (H). Fasting plasma glucose, FPG; Homeostasis model assessment-insulin resistance, HOMA-IR; Homeostasis model of assessment for β cell function, HOMA-B; High-sensitivity C-reactive protein, hs-CRP; Total antioxidant capacity, TAC; Glutathione, GSH; Malondialdehyde, MDA [file 12902_2021_712_MOESM2_ESM.docx]
